# Supplementary material for: Clinical CDK4/6 inhibitors induce selective and immediate dissociation of p21 from cyclin D-CDK4 to inhibit CDK2
Source: Nat Commun. 2021 Jun 7;12:3356. doi: 10.1038/s41467-021-23612-z (PMC8184839; doi:10.1038/s41467-021-23612-z)
Supplement: Supplementary file 1 — Supplementary Information [file 41467_2021_23612_MOESM1_ESM.pdf]

Supplementary Information for

Clinical CDK4/6 inhibitors induce selective and immediate dissociation of p21 from cyclin D-CDK4 to inhibit CDK2

Lindsey R. Pack[1], Leighton H. Daigh[1], Mingyu Chung[1], and Tobias Meyer[1*]

These authors contributed equally: Lindsey R. Pack, Leighton H. Daigh

[1] Department of Chemical and Systems Biology, Stanford University, Stanford, CA

This PDF file includes:
Figs. S1 to S4
Supplementary Table 1

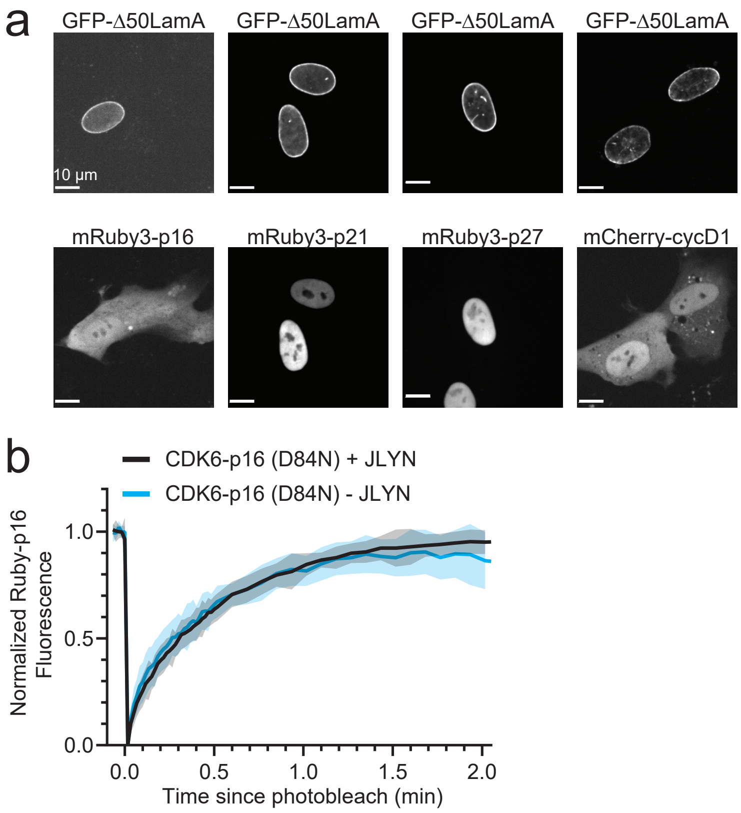

Supplementary Figure 1

Supplementary Fig. 1: p16 (D84N) recovery is not affected by cytoskeleton-stabilizing drugs.
a, Confocal fluorescence images of RPE-1 cells co-transfected with GFP-Δ50 lamin A and mRuby3-p16, mRuby3-p21, mRuby3-p27, or mCherry-cyclin D1 **b,** Comparison of the fluorescence recovery time courses mRuby3-p16 (D84N) from CDK6-GFP-Δ50 lamin A after photobleaching of mRuby3-p16 (D84N) with (n = 23, data from Fig. 1g) and without (n = 18 cells, 2 biological replicates) cytoskeleton-stabilizing drugs. JLYN - jasplakinolide, latrunculin B, Y27631, nocodazole. FRAP curves show mean ± s.d. Source data are provided as a Source Data file.

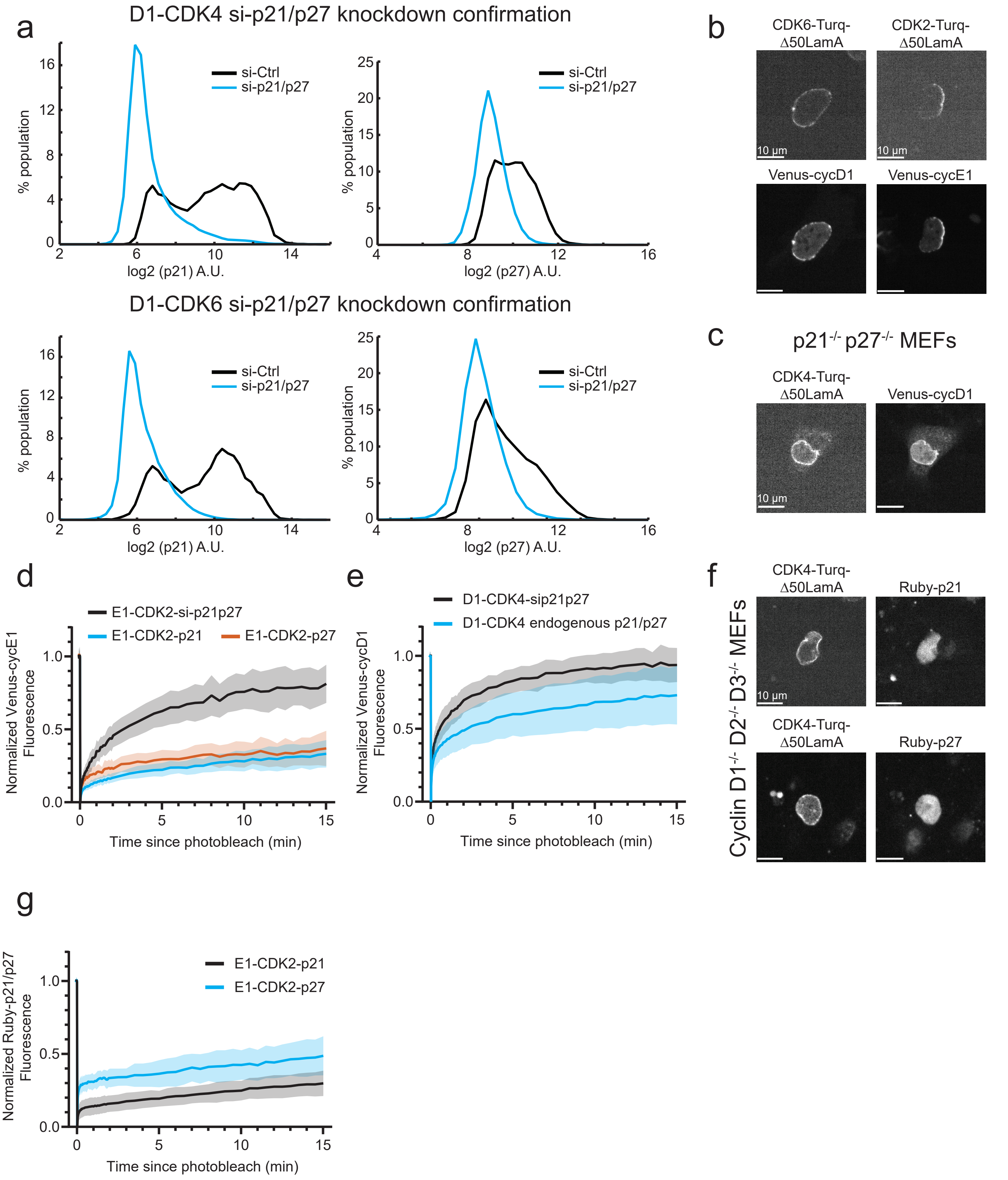

Supplementary Figure 2

Supplementary Fig. 2: cyclin E1-CDK2 is stabilized by p21 and p27.
a, Immunofluorescence validation of p21 and p27 co-knockdown using control or p21/p27 targeting siRNA. Representative histogram in CDK4-complex (si-Ctrl n=16,372 cells, si-p21/p27 n=82,386 cells) and CDK6-complex (si-Ctrl n=14,420 cells, si-p21/p27 n=61,149 cells) experiments from one of three biological replicates. **b,** Confocal fluorescence images of enrichment of mVenus-cyclin D1 and mVenus-cyclin E1 at the nuclear periphery of CDK6-mTurq-Δ50 lamin A and CDK2-mTurq-Δ50 lamin A expressing cells, respectively. **c**, Confocal fluorescence images of p21$^{-/-}$ p27$^{-/-}$ MEFs transfected with CDK4-mTurq-Δ50 lamin A and mVenus-cyclin D1 **d**, mVenus-cyclin E1 FRAP curves in CDK2-mTurquoise-Δ50 lamin A complexes comparing cells depleted of p21 and p27 (n = 18; data from Fig. 2b) to cells co-transfected with mRuby3-p21 (n = 25 cells, 2 biological replicates) or mRuby3-p27 (n = 16 cells, 2 biological replicates). **e,** mVenus-cyclin D1 FRAP curves in cyclinD1-CDK4 complexes comparing mVenus-cyclin D1 recovery following siRNA-mediated knockdown of p21 and p27 (n = 26 cells, 3 biological replicates; replotted from **2b**) to untreated cells with endogenous levels of p21 and p27 (n = 19, 2 biological replicates) **f,** Representative confocal fluorescence images of cyclin D1$^{-/-}$ D2$^{-/-}$ D3$^{-/-}$ MEFs transfected with CDK4-mTurq-Δ50 lamin A and mRuby3-p21 or mRuby3-p27. No enrichment of the mRuby3 signal was observed at the nuclear periphery. **g**, mRuby3-p21 (n = 17 cells, 2 biological replicates) or mRuby3-p27 (n = 10 cells, 2 biological replicates) FRAP curves in mVenus-cyclin E1 and CDK2-mTurquoise-Δ50 lamin A complexes. FRAP curves show mean ± s.d. Source data are provided as a Source Data file.

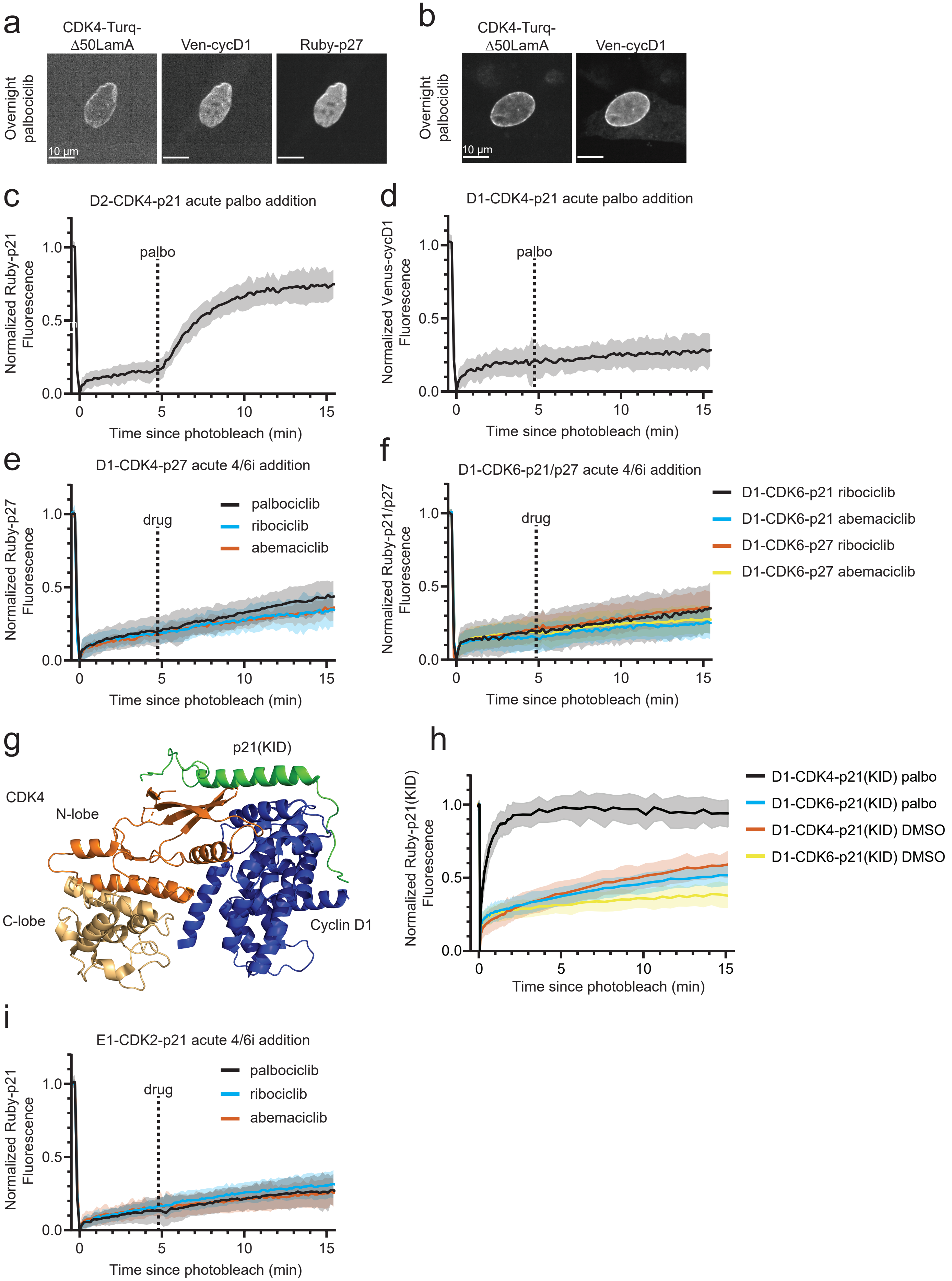

Supplementary Figure 3

Supplementary Fig. 3: Acute treatment with clinical CDK4/6 inhibitors does not affect CIP binding in D1-CDK4-p27, CDK6, or CDK2 complexes.

a, Confocal fluorescence images of mRuby3-p27 and mVenus-Cyclin D1 binding CDK4-mTurquoise-Δ50 lamin A after overnight incubation in palbociclib. **b**, Confocal fluorescence images of mVenus-Cyclin D1 binding CDK4-mTurquoise-Δ50 lamin A after overnight incubation in palbociclib with p21 and p27 siRNA-mediated co-knockdown. **c**, mRuby3-p21 fluorescence recovery in cyclin D2-CDK4-p21 complexes during acute addition of CDK4/6 inhibitor palbociclib (6 μM; n = 13 cells cells, 2 biological replicates). Photobleaching was performed and recovery of the mRuby3-p21 fluorescence signal was measured. At the indicated timepoint, palbociclib was acutely added and continued measurement of mRuby3-p21 fluorescence recovery was performed. **d**, mVenus-cyclin D1 fluorescence recovery in cyclin D1-CDK4-p21 complexes during acute addition of CDK4/6 inhibitor palbociclib (6 μM; n = 19 cells, 3 biological replicates). **e**, mRuby3-p27 fluorescence recovery in cyclin D1-CDK4-p27 complexes during acute addition of the CDK4/6 inhibitors palbociclib (6 μM; n = 12 cells, 3 biological replicates), ribociclib (6 μM; n = 10 cells, 2 biological replicates), or abemaciclib (6 μM; n = 10 cells, 2 biological replicates). **f**, mRuby3-p21 or mRuby3-p27 fluorescence recovery in cyclin D1-CDK6-p21/p27 complexes during acute addition of the CDK4/6 inhibitors. mRuby3-p21 recovery following ribociclib treatment (6 μM; n = 11 cells, 2 biological replicates) or abemaciclib (6 μM; n = 10 cells, 2 biological replicates). mRuby3-p27 recovery following ribociclib treatment (6 μM; n = 15 cells, 2 biological replicates) or abemaciclib (6 μM; n = 16 cells, 3 biological replicates). **g,** Crystal structure of cyclin D1-CDK4-p21KID complex generated from data collected by Guiley *et al*. **h,** FRAP time courses of mRuby3-p21KID in cyclin D1-CDK4-p21KID or cyclin D1-CDK6-p21KID complexes in cells that were maintained in 6 μM palbociclib or DMSO overnight and throughout the photobleaching experiment (cyclin D1-CDK4-p21KID palbociclib n = 14 cells, 2 biological replicates, cyclin D1-CDK6-p21KID palbociclib n = 12 cells, 2 biological replicates, cyclin D1-CDK4-p21KID DMSO n=16 cells, 2 biological replicates, cyclin D1-CDK6-p21KID DMSO n=14, 2 biological replicates). **i**, mRuby3-p21 fluorescence recovery in cyclin E1-CDK2-p21 complexes during acute addition of the CDK4/6 inhibitors palbociclib (6 μM; n = 13 cells, 2 biological replicates), ribociclib (6 μM; n = 18 cells, 2 biological replicates), or abemaciclib (6 μM; n = 13 cells, 2 biological replicates). FRAP curves show mean ± s.d. palbo - palbociclib. Source data are provided as a Source Data file.

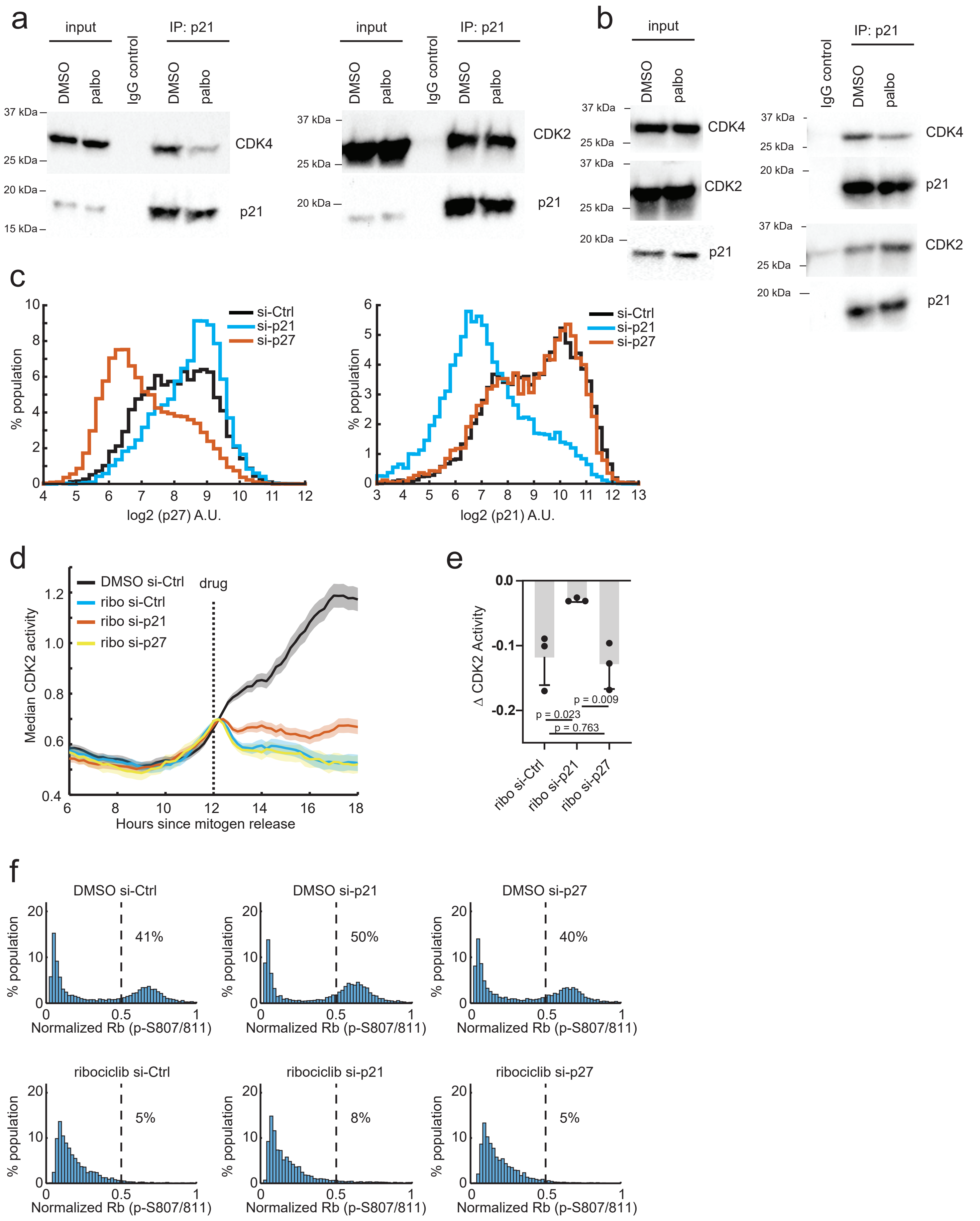

Supplementary Figure 4

Supplementary Fig. 4: Ribociclib indirectly inhibits CDK2 activity via p21 displacement.
a, Asynchronously cycling MCF7 cells were treated with DMSO or palbociclib (6 μM). Immunoprecipitation of p21 was then performed and the amount of p21-bound CDK4 or CDK2 was determined by immunoblotting. **b**, Same analysis as in 4a, but from asynchronously cycling T-47D cells. **c**, Immunofluorescence validation of p21 and p27 knockdown in MCF-10A cells using control, p21, or p27 targeting siRNA. Representative histogram from one of three biological replicates. p27 levels were measured in cells maintained in starvation media. p21 levels were measured in cells 24 hours after serum release. p21 stain: si-Ctrl n=8,327cells, si-p21 n=10,968 cells, si-p27 n=8,979 cells; p27 stain: si-Ctrl n=9,760 cells, si-p21 n=9,122 cells, si-p27 n=10,262 cells. **d**, Median live-cell CDK2 activity traces in ribociclib-treated MCF-10A cells. Cells were first serum starved and immediately treated with the indicated siRNA and maintained in starvation media for another 48 hours. Cells were then restimulated with mitogens and live-cell imaging of CDK2 activity was performed. 12 hours after restimulation, DMSO or ribociclib (3 μM) was acutely added and imaging was continued. Cells were gated for those with CDK2 activity levels between 0.65 and 0.75 at the time of drug addition that had not yet entered S phase, as determined by the FUCCI APC/C^{CDH1} fluorescent reporter. Shaded areas indicate 95% confidence intervals. Data are from one experiment representative of three biological replicates. DMSO si-Ctrl condition uses same data as in Fig. 4b. DMSO si-Ctrl n=281 cells, ribo si-Ctrl n=164 cells, ribo si-p21 n=221 cells, ribo si-p27 n=122 cells. **e**, Bar graph showing average drop in CDK2 activity following addition of ribociclib in si-Ctrl, si-p21, and si-p27 cells, comparing the difference in CDK2 activity at the time of drug spike and the average activity in the last hour of the time course. The results of three biological replicates are shown for each condition (mean ± s.d). One of the replicates is from data shown in (**d**). Replicate 1: DMSO si-Ctrl n=281 cells, ribo si-Ctrl n=164 cells, ribo si-p21 n=221 cells, ribo si-p27 n=122 cells; Replicate 2: DMSO si-Ctrl n=119 cells, ribo si-Ctrl n=55 cells, ribo si-p21 n=113 cells, ribo si-p27 n=127 cells; Replicate 3: DMSO si-Ctrl n=99 cells, ribo si-Ctrl n=193 cells, ribo si-p21 n=366 cells, ribo si-p27 n=183 cells. Statistical analysis was performed using a two-tailed unpaired Student's t-test. **f**, Histogram of MCF-10A cells prepared as in (**d**). Cells were fixed 15 minutes after addition of either DMSO or ribociclib (3 μM) and stained for Rb (phospho-S807/811) and total Rb (phosphosite-independent antibody). Cells were gated for those with CDK2 activity < 0.6 and no S-phase entry, as determined by FUCCI APC/C^{CDH1} fluorescent reporter. For each cell, nuclear intensity of p-S807/811 Rb and total Rb was measured and a ratio of the intensities was calculated. Cells were then normalized to the maximum single-cell ratio and plotted. Percentage of cells with a ratio greater than 0.5, representing cells with hyper-phosphorylated Rb, was calculated for each condition. DMSO si-Ctrl condition uses same data as in Fig. 4f. DMSO si-Ctrl n=5204 cells, DMSO si-p21 n=4572 cells, DMSO si-p27 n=4937 cells, ribo si-Ctrl n=3879 cells, ribo si-p21 n=3833 cells, ribo si-p27 n=3926 cells. Plotted data are representative of three biological replicates. palbo - palbociclib, ribo - ribociclib. Source data are provided as a Source Data file.

Supplementary Table 1

Plasmid	Primer name	Primer Sequence (5' => 3')	Use
CDK4-EGFP-Δ50 lamin A	EGFP-Lamin For	GGATGAAGGTAATCCGGAGGGTGCAGCAGGATCCATGGTGAGCAAGGGCGAGGAG	PCR
	EGFP-Lamin Rev	ctagagtcgcggccgctttacttttacatgatgctgcagttctgggg	PCR
	CDK4 For	cggactcagatctcgagctcagccaccATGGCTACCTCTCGATATGAGCCAGTG	PCR
	CDK4 Rev	CTCGCCCTTGCTCACCATGGATCCTGCTGCACCCTCCGGATTACCTTCATCCTTATGT	PCR
CDK6-EGFP-Δ50 lamin A	EGFP-Lam For	CCTCGGAGCTGAATACAGCCGGTGCAGCAGGATCCatggtgagcaagggcgaggag	PCR
	EGFP-Lam Rev	ctagagtcgcggccgctttacttttacatgatgctgcagttctggggg	PCR
	CDK6 For	ggactcagatctcgagctcaagctgccaccATGGAGAAGGACGG	PCR
	CDK6 Rev	ctcctcgcccttgctcaccatGGATCCTGCTGCACCGGCTGTATTCAGCTCCGAGGTG	PCR
CDK6-mTurq-Δ50 lamin A	mTurq-Lam For	CCTCGGAGCTGAATACAGCCGGTGCAGCAGGATCCatggtgagcaagggcgaggag	PCR
	mTurq Rev	gctcgagatctgagtccggacttgtacagctcgtccatgccgag	PCR
mRuby-p16	mRuby For	ggactcagatctcgagctcaagcttgccaccatggtgtctaagggcgaagagctg	PCR
	mRuby Rev	CCAGCGGCTGGTTCggatccgtcgaccttgtacagctcgtccatgcc	PCR
	p16 For	ggtggcatggacgagctgtacaaggtcgacggatccGAACCAGCCGCTG	PCR
	p16 Rev	gattatgatctagagtcgcggccgctTCAGTCTGGGATGTCAGATGGTCCTTCAGCGG	PCR
mRuby-p16 (D84N)	p16D84N For	CCAGACCAGTACACAACGCCGCTAGGGAG	PCR mutagenesis
	p16D84N Rev	CTCCCTAGCGGCGTTGTGTACTGGTCTGG	PCR mutagenesis
	p16 For	ggtggcatggacgagctgtacaaggtcgacggatccGAACCAGCCGCTG	PCR
	p16 Rev	gattatgatctagagtcgcggccgctTCAGTCTGGGATGTCAGATGGTCCTTCAGCGG	PCR
mRuby-p21	p21 For	GGCATGGACGAGCTGTACAAGtcgggcggatccATGTCTGAACCCGCTGGCG	PCR
	p21 Rev	ggctgattatgatctagagtcgcggccgctTCAGGGCTTTCGTTTGGAAAATATGAGGC	PCR

mRuby-p27	p27 For	GGCATGGACGAGCTGTACAAGtcgggcggatccATGTCAAACGTGCGAGTGTC	PCR
	p27 Rev	ggctgattatgatctagagtcgcggccgctTCAAGTTTGACGTCTTCTGAGGCC	PCR
mVenus-Cyclin D1	mVenus For	GGACTCAGATCTCGAGCTCAAGCTTGCCACCATGGTGAGCAAGGGCGAGG	PCR
	mVenus Rev	CCCAGAACCGCCAGACCCACTACCTCCTGACTTGTACAGCTCGTCCATGCCG	PCR
	Cyclin D1 For	ggactcagatctcgagctcaagcttgccaccATGGTGAGCAAGGGCGAGG	PCR
	Cyclin D1 Rev	gattatgatctagagtcgcggccgctttaAATATCGACATCGCGGACATCG	PCR
mVenus-Cyclin E1	Cyclin E1 For	GGCATGGACGAGCTGTACAAGtcgggcggatccccgagggagcgcagggag	PCR
	Cyclin E1 Rev	ggctgattatgatctagagtcgcggccgcttcacgccatttccggcccg	PCR
CDK4-mTurq-Δ50 lamin A	CDK4 For	atccgctagcgctaccggactcagatctcgagctcaagctgccaccATGgctacctctcgatatgagc	PCR
	CDK4 Rev	CTCGCCCTTGCTCACCATGGATCCTGCTGCACCCTCCGGATTACCTTCATCCTTATGT	PCR
CDK2-mTurq-Δ50 lamin A	CDK2 For	atccgctagcgctaccggactcagatctcgagctcaagctgccaccatggagaacttccaaaaggt	PCR
	CDK2 Rev	ctcctcgcccttgctcaccatGGATCCTGCTGCACCgagtcgaagatggggtactgg	PCR
mRuby-p21(14-85)	p21 KID For	acgagctgtacaagtcgggcggatccATGGGCTCCAAAGCCTGTCG	PCR
	p21 KID Rev	attatgatctagagtcgcggccgctTCAACCGGTGGGGAGATAGAG	PCR
