# Supplementary material for: Clinical CDK4/6 inhibitors induce selective and immediate dissociation of p21 from cyclin D-CDK4 to inhibit CDK2
Source: Nat Commun. 2021 Jun 7;12:3356. doi: 10.1038/s41467-021-23612-z (PMC8184839; doi:10.1038/s41467-021-23612-z)
Supplement: Supplementary file 3 — Description of Additional Supplementary Files [file 41467_2021_23612_MOESM3_ESM.docx]

Description of Additional Supplementary Files

Title: Supplementary Movie 1

Description: Rapid dissociation of p16 (D84N) from CDK6-GFPΔ50 lamin A. Cells were co-transfected with plasmids encoding mRuby3-p16 (D84N) and CDK6-GFP-Δ50 lamin A. Photobleaching of mRuby3-p16 (D84N) at the nuclear periphery was performed and recovery of the mRuby3 fluorescent signal was monitored. Time stamp hh:mm:ss; Scale bar, 10 μm.

Title: Supplementary Movie 2

Description: Palbociclib immediately dissociates p21 from cyclin D1-CDK4 complexes. Cells were co-transfected with plasmids encoding mVenus-cyclin D1, CDK4- GFP-Δ50 lamin A, and mRuby3-p21. Photobleaching of mRuby3-p21 at the nuclear periphery was performed and recovery of the mRuby3 fluorescent signal was monitored. DMSO or Palbociclib (6 μM) were acutely added at the indicated timepoint and imaging was continued. Time stamp mm:ss; Scale bar, 5 μm.
