# Supplementary material for: Clinical CDK4/6 inhibitors induce selective and immediate dissociation of p21 from cyclin D-CDK4 to inhibit CDK2
Source: Nat Commun. 2021 Jun 7;12:3356. doi: 10.1038/s41467-021-23612-z (PMC8184839; doi:10.1038/s41467-021-23612-z)
Supplement: Supplementary file 6 — Reporting Summary [file 41467_2021_23612_MOESM6_ESM.pdf]

nature research

Corresponding author(s): Tobias Meyer

Last updated by author(s): Apr 29, 2021

Reporting Summary

Nature Research wishes to improve the reproducibility of the work that we publish. This form provides structure for consistency and transparency in reporting. For further information on Nature Research policies, see our Editorial Policies and the Editorial Policy Checklist.

Statistics

For all statistical analyses, confirm that the following items are present in the figure legend, table legend, main text, or Methods section.

n/a	Confirmed	
☐	☒	The exact sample size (n) for each experimental group/condition, given as a discrete number and unit of measurement
☐	☒	A statement on whether measurements were taken from distinct samples or whether the same sample was measured repeatedly
☐	☒	The statistical test(s) used AND whether they are one- or two-sided<br>*Only common tests should be described solely by name; describe more complex techniques in the Methods section.*
☒	☐	A description of all covariates tested
☒	☐	A description of any assumptions or corrections, such as tests of normality and adjustment for multiple comparisons
☐	☒	A full description of the statistical parameters including central tendency (e.g. means) or other basic estimates (e.g. regression coefficient) AND variation (e.g. standard deviation) or associated estimates of uncertainty (e.g. confidence intervals)
☐	☒	For null hypothesis testing, the test statistic (e.g. F, t, r) with confidence intervals, effect sizes, degrees of freedom and P value noted<br>*Give P values as exact values whenever suitable.*
☒	☐	For Bayesian analysis, information on the choice of priors and Markov chain Monte Carlo settings
☒	☐	For hierarchical and complex designs, identification of the appropriate level for tests and full reporting of outcomes
☒	☐	Estimates of effect sizes (e.g. Cohen's d, Pearson's r), indicating how they were calculated

Our web collection on statistics for biologists contains articles on many of the points above.

Software and code

Policy information about availability of computer code

Data collection	MetaExpress version 6.1, a software provided by Molecular Devides, was used for acquisition of microscopy images on the ImageExpress Micro. Slidebook 6.13, a software provided by 3i, was used for acquisition of confocal microscopy images.
Data analysis	Matlab code was used for image and data analysis, a repository of cell segmenting and tracking code has been cited. Other data was analyzed in Slidebook 6.13 and Excel365 and plotted and statistically analyzed using GraphPad Prism v8.0.

For manuscripts utilizing custom algorithms or software that are central to the research but not yet described in published literature, software must be made available to editors and reviewers. We strongly encourage code deposition in a community repository (e.g. GitHub). See the Nature Research guidelines for submitting code & software for further information.

Data

Policy information about availability of data

All manuscripts must include a data availability statement. This statement should provide the following information, where applicable:

- Accession codes, unique identifiers, or web links for publicly available datasets
- A list of figures that have associated raw data
- A description of any restrictions on data availability

The authors declare that data supporting the findings of this study are available within the paper and its supplementary information files. Source data are provided with this paper.

Field-specific reporting

Please select the one below that is the best fit for your research. If you are not sure, read the appropriate sections before making your selection.

- [x] Life sciences
- [ ] Behavioural & social sciences
- [ ] Ecological, evolutionary & environmental sciences

For a reference copy of the document with all sections, see nature.com/documents/nr-reporting-summary-flat.pdf

Life sciences study design

All studies must disclose on these points even when the disclosure is negative.

Sample size	No sample-size calculation was performed. Sample size was determined to be adequate based on the magnitude and consistency of the measurable differences between groups. All experiments were repeated on multiple days to ensure reproducibility. For experiments where significance was calculated, n of at least 3 was predetermined to enable adequate statistical testing.
Data exclusions	No data was excluded in this study, although during quantification in our MATLAB code certain cells are gated out as follows. For live-cell imaging cells were excluded if they could not be distinguished from a neighboring cell or were not tracked for a significant period of time in the experiment.
Replication	Attempts at replication were successful, and all experiments were repeated on multiple days. Number of times experiments were repeated can be found in the figure legends. Experiments where significance were derived have at least n=3.
Randomization	In cell line experiments, no randomization was required because the experimental cells came from the same source and were tested identically with the variable treatment. Cell lines were used for the entirety of this study; no other sample type that would require randomization was used in this study.
Blinding	Blinding was not done. All experiments were performed at least twice to ensure reproducibility and examined by multiple authors independently via unbiased analysis pipeline. Blinding was not considered based upon experience and similar published studies.

Reporting for specific materials, systems and methods

We require information from authors about some types of materials, experimental systems and methods used in many studies. Here, indicate whether each material, system or method listed is relevant to your study. If you are not sure if a list item applies to your research, read the appropriate section before selecting a response.

Materials & experimental systems

n/a	Involved in the study	
☐	☒	Antibodies
☐	☒	Eukaryotic cell lines
☒	☐	Palaeontology and archaeology
☒	☐	Animals and other organisms
☒	☐	Human research participants
☒	☐	Clinical data
☒	☐	Dual use research of concern

Methods

n/a	Involved in the study	
☒	☐	ChIP-seq
☒	☐	Flow cytometry
☒	☐	MRI-based neuroimaging

Antibodies

Antibodies used	CDK4 (Rabbit monoclonal, Abcam, ab108357, EPR4513-32-7), p21 (Rabbit polyclonal, CST, 2947S, 12D1), CDK4 (Mouse monoclonal, Santa Cruz, sc-56277, DCS-31), p21 (Mouse monoclonal, Fisher, BDB556430, SX118), FLAG (Mouse monoclonal, Sigma, F3165, M2), CDK2 (Mouse monoclonal, Origene, TA502915, OTI2A5), GST (Mouse monoclonal, Santa Cruz, sc-138, B-14), Rb (p-807/811) (Rabbit monoclonal, CST, 8516, D20B12), Rb (Mouse monoclonal, CST, 9309, 4H1), p27 (Rabbit monoclonal, CST, 3686, D69C12), p27 (Mouse monoclonal, CST, 3698, SX53G8.5), Alexa Fluor goat secondary antibodies (Goat Anti-Rabbit 568,ThermoFisher, A-11036),(Goat Anti-Rabbit 647,ThermoFisher, A32733), (Goat Anti-Mouse 568, ThermoFisher, A-11004), and (Goat Anti-Mouse 647, ThermoFisher, A21235), HRP conjugated secondary antibodies (Anti-rabbit IgG, CST, 7074) and (Anti-mouse IgG, CST, 7076), Normal Rabbit IgG (CST, 2729S)
Validation	Antibodies validated through siRNA knockdown and cellular localization using immunofluorescence: CDK4 (Abcam, ab108357), p21 (CST, 2947S), p21 (Fisher, SX118), Rb (p-807/811) (CST, 8516), Rb (CST, 9309), p27 (CST, 3686), p27 (CST, 3698)<br><br>Antibodies validated by Western Blots, including via either band size and controls in IPs: CDK4 (Abcam, ab108357), p21 (CST, 2947S), CDK4 (Santa Cruz, sc-56277), p21 (Fisher, SX118), FLAG (Sigma, F3165), CDK2 (Origene, OTI2A5, GST (Santa Cruz, sc-138)<br><br>Statements on manufacturer's website or comments about citations where relevant:<br>CDK4 (Abcam, ab108357): "Knockout validated" in both Western and immunofluorescence assays.

p21 (CST, 2947S): "p21 Waf1/Cip1 (12D1) Rabbit mAb detects endogenous levels of total p21 protein. The antibody does not cross-react with other CDK inhibitors."
p21(Mouse monoclonal, Fisher, BDB556430, SX118): "The epitope has been mapped to the last 20 amino acids (residues 145-164) of human p21, one of the most conserved regions between human and mouse p21. Reaction of the antibody with overlapping peptides fragments suggest that the epitope may be further mapped to residues 145-156 (TSMTDFYHSKRR). This sequence overlaps with the sequence of p21 (KRRQTSMTDFYH) which is responsible for the specific interaction of p21 with PCNA. This antibody is routinely tested by western blot analysis. Other applications were tested at BD Biosciences Pharmingen during antibody development or reported in the literature only."
CDK4 (Santa Cruz, sc-56277): "non cross-reactive with other CDK types"
FLAG (Sigma, F3165): "This monoclonal antibody is produced in mouse and recognizes the FLAG sequence at the N-terminus, Met N-terminus, and C-terminus. The antibody is also able to recognize FLAG at an internal site. M2, unlike M1 antibody is not Calcium dependent."
GST (Santa Cruz, sc-138): "raised against the 26 kDa GST specific domain of a fusion protein encoded by a pGEX.3X recombinant vector" and "GST Antibody (B-14) is recommended for detection of GST fusion proteins and glutathione-S-transferase (GST) of Schistosoma japonicum origin by WB and IP"
Rb (p-807/811) (CST, 8516):Phospho-Rb (Ser807/811) (D20B12) XP® Rabbit mAb recognizes endogenous levels of Rb protein only when phosphorylated at Ser807, Ser811, or at both sites. This antibody does not cross-react with Rb phosphorylated at Ser608.
Rb (CST, 9309): "Rb (4H1) Mouse mAb detects endogenous levels of total Rb protein. The antibody does not cross-react with the Rb homologues p107 or p130, or with other proteins."

Eukaryotic cell lines

Policy information about cell lines

Cell line source(s)	hTERT RPE-1 cells (acquired directly from ATCC, #CRL-4000), MCF-10A cells (acquired directly from ATCC, #CRL-10317, RRID:CVCL_0598, human female), p21-/-p27-/- MEFs (generated in the lab of Charles Sherr), cyclin D1-/-D2-/-D3-/- MEFs (generated in the lab of Peter Sicinski), MCF7 cells (Fan lab from ATCC, HTB-22), T-47D (Yang lab from ATCC, HTB-133)
Authentication	MCF-10A were validated by RNA-seq. Other cell lines were not validated.
Mycoplasma contamination	hTERT RPE-1, MCF-10A, MCF7 cells were mycoplasma negative. Other cell lines were not tested.
Commonly misidentified lines (See ICLAC register)	No commonly misidentified lines were used in this study.
